# Flattened microvessel independently predicts poor prognosis of patients with non-small cell lung cancer

**DOI:** 10.18632/oncotarget.15617

**Published:** 2017-02-22

**Authors:** Luo Fang, Ying He, Yinghui Tong, Luying Hu, Wenxiu Xin, Yujia Liu, Like Zhong, Yiwen Zhang, Ping Huang

**Affiliations:** ^1^ Laboratory of Clinical Pharmacy, Zhejiang Cancer Hospital, Hangzhou, Zhejiang, China; ^2^ Zhejiang Key Laboratory of Diagnosis and Treatment Technology on Thoracic Oncology (Lung and Esophagus), Hangzhou, Zhejiang, China

**Keywords:** flattened microvessel, aspect ratio, microvessel abnormality, prognosis, non-small cell lung cancer (NSCLC)

## Abstract

Angiogenesis plays an essential role in improving tumor progression, whereas, its value in prognosis predicting remains controversial, especially in non-small cell lung cancer (NSCLC). Most recently, microvessel pattern has been raised as a novel prognosis factor. In this study, flattened microvessel, evaluated by tumor microvessel aspect ratio (TMAR), was conducted as a prognostic factor in NSCLC patients. A total of 100 patients with NSCLC were retrospectively reviewed. Microvessel in tumor was visualized by immunochemistry staining and then TMAR was determined. The prognostic role of TMAR was evaluated by univariate and multivariate analysis. Most of intratumor microvessels were flattened with a median TMAR of 3.65 (range, 2.43 - 6.28). Patients were stratified into high TMAR group (TMAR ≥ 3.6) and low TMAR group (TMAR < 3.6). Compared with subpopulation with low TMAR, high TMAR had significantly high risk of cancer-related death (univariate analysis: HR = 5.06, 95% CI: 2.44-10.47, *p*<0.001; multivariate analysis: HR = 4.53, 95% CI: 1.70-12.06, *p*=0.002).

In conclusion, the results of our study demonstrate that flattened microvessel in tumor tissue is a promising prognosis predictor of NSCLC patients.

## INTRODUCTION

Lung cancer is a worldwide common cancer characterized as most diagnosed human malignancies and leading cause of cancer-related deaths worldwide, of which non-small cell lung cancer (NSCLC) accounts for about 85% [1]. NSCLC responds poorly to therapy, the 5-year survival rate remains about 15% [2, 3]. The dramatic progression and poor prognosis of NSCLC is attributed to several “hallmarks”, including angiogenesis [4].

Tumor-associated neovasculature plays a vital role in pathogenesis, progression, invasion and metastasis of NSCLC by addressing the need of oxygen and nutrient [5, 6]. Theoretically, the properties of tumor angiogenesis, which are popularly represented by microvessel density (MVD) or microvessel area (MVA), are expected to be promising factors of patient outcome [7–10]. However, the role of either MVD or MVA in the prognosis predicting of NSCLC is controversial [11–13].

Most recently, the profile of microvessel pattern was raised as a novel factor of prognosis prediction [14–20]. As reported, abnormal microvessel was associated with reduction of blood flow, hypoxia microenvironment, increase of interstitial fluid pressure (IFP) and ultimately tumor progression [21–24]. Microvessel with abnormal pattern, such as branched vessel, vascular garlands, glomeruloid vascular formations, and irregular outline, was characterized by an algorithm and evaluated as an independent prognosis factor in a few studies about astrocytic tumor [14, 16, 18] and neuroblastoma [17]. Apart from aforementioned bizarre pattern, there are many common deformities of microvessel observed in tumor, especially flattened vessel. In addition, the microvessel pattern in patients with other solid tumors is rarely reported. Thus, this study was focused on the flattened microvessel of patients with NSCLC. We characterized deformation of flattened microvessel by tumor microvessel aspect ratio (TMAR, defined as ratio of long axis to short axis length of microvessel) and assessed the correlation between TMAR and survival outcome to determine whether TMAR is an independent prognostic factor for NSCLC patients.

## RESULTS

### Patients and clinical data

A total of 100 patients presented as adenocarcinoma (54.0%), squamous (43.0%) or others (3.0%) were included in this retrospective study. Median age was 59 years and 74 patients (74.0%) were male. Most patients were on early stage (n = 71). Clinicopathologic characteristics of patients were described in Table 1. Only one patient received anti-angiogenesis therapy (Recombinant Human Endostatin Injection, ENDOSTAR ^®^, Simcere Pharmaceutical, Nanjing, China). The median follow-up time was 51.1 months (range, 45.5 - 60.6 months). None patient was lost to follow-up of overall survival. In terms of recurrence, 8 patients (8.0%) were lost.

**Table 1:** Basic characteristics and TMARs of patients with NSCLC

Clinicopathologic variables	Number (%)	TMAR^a^		
		Low (%) (n = 47)	High (%)(n = 53)	Mean ± SD
Age				
<60	51 (51.0)	22 (46.8)	29 (54.7)	3.84± 0.72
≥60	49 (49.0)	25 (53.2)	24 (45.3)	3.74± 0.75
Gender				
Male	74 (74.0)	35 (74.5)	39 (73.6)	3.8±0.74
Female	26 (26.0)	12 (25.5)	14 (26.4)	3.76±0.74
Smoking history				
Never	33 (33.0)	15 (31.9)	18 (34.0)	3.81±0.78
Prior or current	67 (67.0)	32 (68.1)	35 (66.0)	3.78±0.71
Disease stage				
Early (stage I & II)	71 (71.0)	36 (76.6)	35 (66.0)	3.71±0.67
Advanced (stage III & IV)	29 (29.0)	11 (23.4)	18 (34.0)	3.99±0.84
Tumor histology				
Adenocarcinoma	54 (54.0)	24 (51.1)	30 (56.6)	3.83±0.82
Squamous	43 (43.0)	22 (46.8)	21 (39.6)	3.71±0.62
Others	3 (3.0)	1 (2.1)	2 (3.8)	4.11±0.52
Tumor differentiation				
Poorly	49 (49.0)	22 (46.8)	27 (50.9)	3.92±0.86
Moderately	47 (47.0)	23 (48.9)	24 (45.3)	3.65±0.57
Well	1 (1.0)	1 (2.1)		

^a^ TMAR, tumor microvessel aspect ratio; Low TMAR < 3.6, High TMAR ≥ 3.6.

### TMAR values in NSCLC tissue and its correlation to clinicopathologic variables

**Figure 1:**
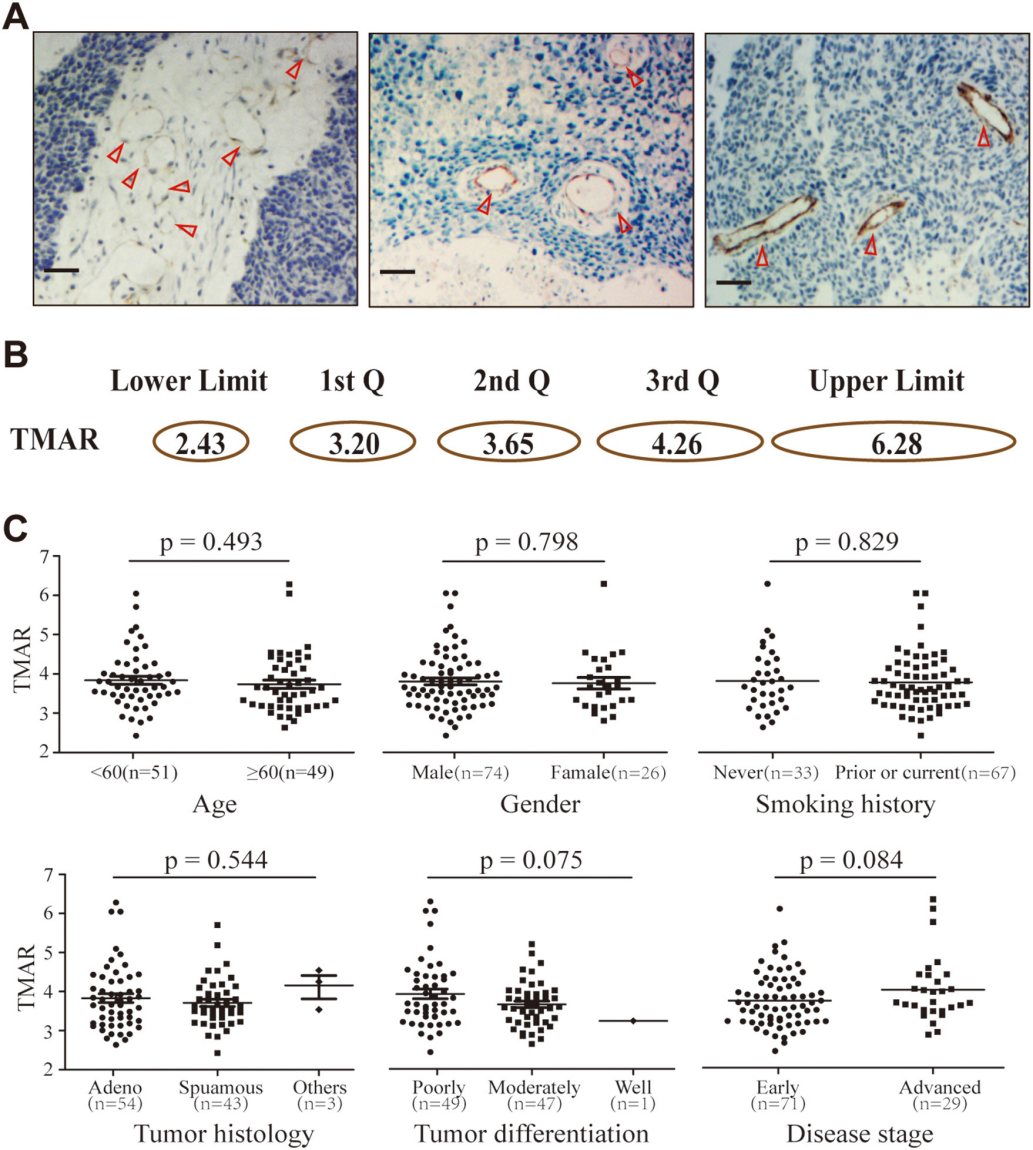
Tumor vascular patterns and TMAR values in NSCLC tissue specimens **A.** Normal (left), slightly flattened (middle) and obviously flattened microvessels (right) positively immune-stained by CD31 (red triangle). Scale bar: 50 μm. **B.** Schematic representation of microvessel with various TMAR value (1^st^ Q: the first quartile value, 2^nd^ Q: the second quartile value, 3^rd^ Q: the third quartile value); **C.** TMAR values stratified by different clinicopathologic factors, including age, gender, smoking history, tumor histology, tumor differentiation, and disease stage.

As shown in Figure 1A, vascular endothelial cells positively expressing CD31 were brown stained. Microvessels were displayed with two patterns, normal round pattern (Figure 1A, left panel), slightly flattened pattern (Figure 1A, middle panel) and highly flattened pattern (Figure 1A, right panel). The mean TMAR of individual patients ranged from 2.43 to 6.28 with a median value of 3.65. The 1^st^, 2^nd^, and 3^ird^ quartile values of TMAR were 3.20, 3.65, and 4.26, respectively. Schematic representation of microvessel with various TMAR was presented in Figure 1B. All patients were stratified into two groups, low TMAR group (TMAR < 3.6) and high TMAR group (TMAR ≥ 3.6). For clinicopathologic variables, it was comparable between the patients with low and high TMAR (*p* > 0.05, Supplementary Table 1).

In various subpopulation stratified by different clinicopathologic variables, including age (< 60, and ≥60), gender (male, and female), smoking history (never, and, prior or current), tumor histology (adenocarcinoma, squamous-cell carcinoma, and others), tumor differentiation (poorly, moderately, and well-differentiated), or disease stage (early, and advanced), no significant differences were found for TMARs (*p* > 0.05 for all subgroups) (Figure 1C).

### High TMAR value presented poor outcome

**Figure 2:**
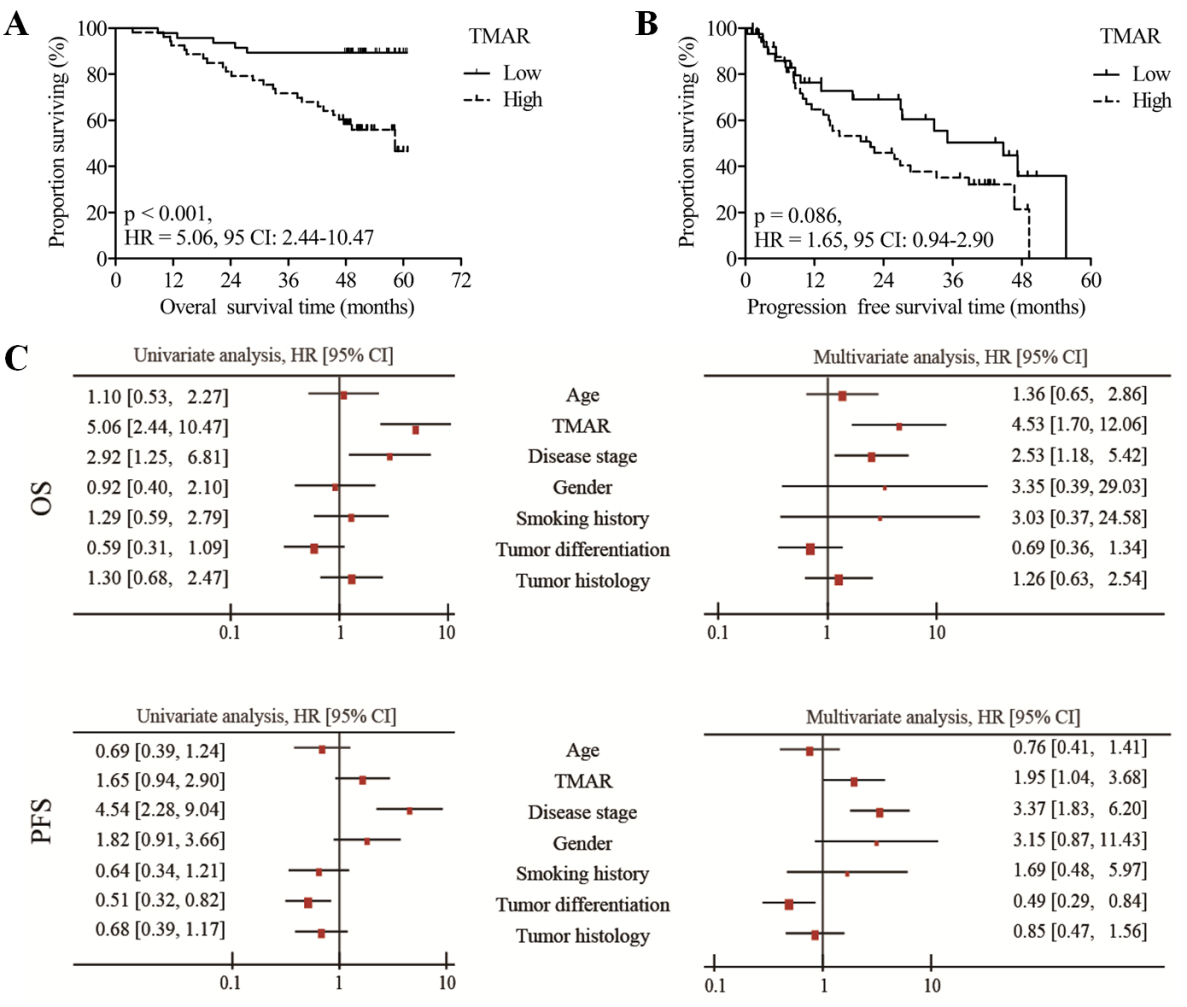
Survival analysis Kaplan-Meier curves of OS **A.** and PFS **B.** for high and low TMAR groups. **C.** Univariate and multivariate analysis of OS and PFS for TMAR and clinicopathologic factors.

During follow-up, 29 of 100 patients (29.0%) had died, and 48 of 92 patients (52.2%) experienced recurrence. As shown in Figure 2A, OS of patients with high TMAR was significantly shorter compared with patients with low TMAR (HR = 5.06, 95% CI: 2.44 - 10.47, *p* < 0.001). Consistently, patients in high TMAR group had an increased risk of recurrence compared with that of patients in low TMAR group (HR = 1.65, 95% CI: 0.94 - 2.90), though a remarkable difference was not detected (*p* = 0.086) (Figure 2B). Apart from high TMAR, advanced disease stage was significantly associated with shorter OS (HR = 2.92, 95% CI: 1.25 - 6.81, *p* = 0.001, left panels of Figure 2C). In terms of PFS, advanced disease stage was found to predict shorter PFS (HR = 4.54, 95% CI: 2.28 - 9.04, *p* < 0.001), while well-differentiated NSCLC was found to correlate with longer PFS (HR = 0.51, 95% CI: 0.32 - 0.82, *p* = 0.041, left panels of Figure 2C).

In multivariate analysis (Figure 2C, right panels), high TMAR was found to be an independent indicator of shorter OS (HR = 4.53, 95% CI: 1.70 - 12.06, *p* = 0.002) and shorter PFS (HR = 1.95, 95% CI: 1.04 - 3.68, *p* = 0.038). Comparably, advanced disease stage predicted a poor survival outcome (OS: HR = 2.53, 95% CI: 1.18 - 5.42, *p* = 0.017 and PFS: HR = 3.37, 95% CI: 1.83 - 6.20, *p* < 0.001). Poorly differentiated disease was found to be associated with shorter PFS (HR = 0.49, 95% CI: 0.29 - 0.84, *p* = 0.010).

## DISCUSSION

Angiogenesis is a hallmark of tumor due to its crucial role in the genesis and progression of tumor [4]. However, accumulated evidences suggested that the value of popular markers of microvessel in tumor, such as MVD and MVA, was controversial [7]. Recently, it was reported that microvessel with abnormal pattern impaired tumor perfusion, induced hypoxia of microenvironment, and reduced drug accumulation in the site of tumor. Therefore, deformed microvessel was expected as a potential marker of patient outcome [15]. In this study, prognostic significance of flattened microvessel in NSCLC was investigated. TMAR was introduced to evaluate deformation for flattened microvessel away from round shape. Of special interest, we found that high TMAR was an independently predictive factor for poor OS and PFS in multivariate analysis.

Microvessel is essential to the progression of tumor by guaranteeing abundant blood perfusion and satisfying the increasing need of oxygen and nutrients. Therefore, the profile of angiogenesis has an impact on clinical outcome in theory. MVD and MVA are proposed as primary and popular markers of tumor angiogenesis and expected to be promising factors of patient outcome prediction. However, the role of MVD or MVA in the prognosis value in cancer patients remains controversial [7, 25]. With the exception being several evidences that described proven correlation between MVD/MVA and patient outcome [26–29], no significant relation was also reported [7, 11, 30]. Alternatively, microvessel pattern was introduced to determine the correlation of perfusion system and patients' outcome.

Currently, a few studies described the prognostic significance of microvessel-morphology changes, including branching capillaries, bizarre vascular pattern and irregular outline in glioblastoma [14, 16] and neuroblastoma [17]. Those abnormal and disorganized patterns cause low tumor perfusion, increased IFP and compromised drug transport [24, 31–34]. In terms of clinical value, Hainfellner and his co-workers conducted an algorithm to evaluate the deformity of vessel consisting of glomeruloid-like, garland-like, or clustered patterns. They disclosed that a high percentage of bizarre microvessels independently predicted poor outcomes in patients with glioblastoma [14, 16]. Similarly, Tadeo defined another deformed vessel by an algorithm based on shape factor, axis, and perimeter and the study found that abnormal microvessels, especially irregular sinusoid vessels, correlated with unfavorable neuroblastic tumors [17]. In addition, most of the studies were focused on brain tumors. In our study, we estimated the deformation degree of flattened microvessel in tumor tissues from NSCLC patients based on the value of TMAR. The deformed microvessel may be attributed to high level of intratumor solid stress. Owing to dramatical growth-rate of tumor in a confined space, high tension of stress is harbored [15, 35]. According to *ex vivo* and *in silico* data, the solid stress is ranged from 35 mmHg to 142 mmHg (4.7-18.9 kPa). The stress was high enough to compress blood vessels [15, 35–37]. Although flattened microvessel was previously outlined at external region of tumors, which may be attributed to the combined effect of radial stress and tensile circumferential stress [35, 38], its role in the prediction of prognosis is rarely reported. In our case, it was disclosed that flattened microvessel significantly linked to poor outcome of NSCLC patients. It could be attributed to the impaired tumor perfusion by shrunken vessel lumen and facilitated tumor-progression by increased solid stress. Compressed blood vessels were narrowed down to incredibly shrinked area compared with normal vessels. It significantly reduced tumor perfusion and caused severe hypoxia in microenvironment, which induced immune-evasion, migration of cancer cell, and even chemotherapeutic resistance [15, 38–42]. In addition, solid stress was proven as a driver of tumor progression, especially through inducing tumorigenesis by activation of the tumorigenic β-catenin pathway [43], enhancing migration by decreasing cell-cell contact, increasing extracellular matrix (ECM) degradation, and ultimately promoting invasion [44–47].

There are also some limits in our study. For the reason that we included both the patients with early stage and advanced stage, the median OS was unable to be obtained. Additionally, more proofs from multi-center studies are needed, in consideration that our results were concluded from a single-center study.

In summary, our findings indicate that flattened microvessel is a promising prognosis factor of patients with NSCLC. A high TMAR independently predicts poor survival outcome. Our results provide a novel insight into the microvessel pattern based prognosis prediction of NSCLC.

## MATERIALS AND METHODS

### Patients

The NSCLC patients who underwent surgical resection at Zhejiang Cancer Hospital between July 2011 and October 2012 were retrospectively reviewed and included when they met all the following eligibility criteria: 1) with histologically diagnosed primary NSCLC; 2) underwent surgical resection as a primary treatment; 3) with available biopsied tumor tissue of primary lesion collected prior to any non-surgery therapy; 4) with full information including clinicopathologic characteristics and survival outcome. The disease stage was classified based on the 7^th^ edition of international staging manual [48]. Tumor histological type and degree of differentiation were determined according to the World Health Organization criteria. For each patient, follow-up was ended up to July 20^th^, 2016. Computed tomography (CT), ultrasonography, magnetic resonance imaging (MRI), and chest radiography were employed to check the recurrence. Tumor specimens were collected from the Tissue Bank of Zhejiang Cancer Hospital. This study was approved by the Ethics Committee of Zhejiang Cancer Hospital and performed in accordance with the Declaration of Helsinki.

### Immunohistochemical staining

Intratumor microvessel was visualized by immunohistochemical staining of CD31. Paraffin-embedded tumor tissues specimens were obtained and cut into consecutive sections (5-μm in thickness). After de-paraffinization and re-hydration, sections underwent target retrieval by incubation with EnVisionTM FLEX Target Retrieval Solution (high pH, pH 9.0, Dako) at 95°C for 20 minutes, followed by blocking non-specific binding sites using 5% BSA solution. Then the sections were incubated with primary antibody of rabbit polyclonal CD31 antibody (dilution: 1:300, Proteintech, Rosemont, USA) at 4°C overnight. Afterwards, they were treated with secondary antibody (Dako) at room temperature for 30 minutes and thus incubated with 3,3′-diaminobenzamine-Tris-HCl solution (50 mmol/L, pH 7.5) containing 0.005% hydrogen peroxide for color development. Finally, the sections were counterstained using hematoxylin.

### TMAR assessment

For TMAR assessment, microvessels positively stained by CD31 within the “hot spot” field were included. The “hot-spot” field was selected according to method previously reported by Vermeulen et al [49]. In brief, an immune-stained section was observed under a low magnification (100 ×) and the field containing the highest density of microvessel was selected. Thereafter, the length of both long axis (*a*) and short axis (*b*) were evaluated under a high magnification (200 ×) and the TMAR was calculated according to the following formula: TMAR = *a*/*b*. And the median value of TMAR was used as a cut-off value to dichotomize patients.

### Statistic analysis

The difference of continuous variables was evaluated with student's t test or analysis of variance (ANOVA) where appropriate, while percentages were provided for categorical variables and compared using chi-square test or Fisher's exact test. Overall survival time (OS) and progression free survival time (PFS) were calculated from the day of first diagnosis of NSCLC until death and any proof of disease progression showed, respectively. The correlation of TMAR and survival outcome of patients was evaluated by both univariate and multivariate analysis. For univariate analysis, both OS and PFS curves were assessed using the Kaplan-Meier model and compared using the Log-rank test. For multivariate analysis, Cox proportional-hazards regression was performed for several clinical variables (age, gender, smoking history, tumor histology, tumor differentiation, and disease stage) to identify the independent contribution of TMAR to patient survival. All analyses with two-sided *p* values (< 0.05) were considered to be statistically significant.

## SUPPLEMENTARY FIGURE AND TABLE



## Abbreviations

ANOVA: analysis of variance
CT: computed tomography
ECM: extracellular matrix
IFP: interstitial fluid pressure
MRI: magnetic resonance imaging
MVA: microvessel area
MVD: microvessel density
NSCLC: non-small cell lung cancer
OS: overall survival time
PFS: progression free survival time
TMAR: tumor microvessel aspect ratio
