# Supplementary material for: Flattened microvessel independently predicts poor prognosis of patients with non-small cell lung cancer
Source: Oncotarget. 2017 Feb 22;8(18):30092–9. doi: 10.18632/oncotarget.15617 (PMC5444728; doi:10.18632/oncotarget.15617)
Supplement: Supplementary file 1 [file oncotarget-08-30092-s001.pdf]

Flattened microvessel independently predicts poor prognosis of patients with non-small cell lung cancer

SUPPLEMENTARY FIGURE AND TABLE

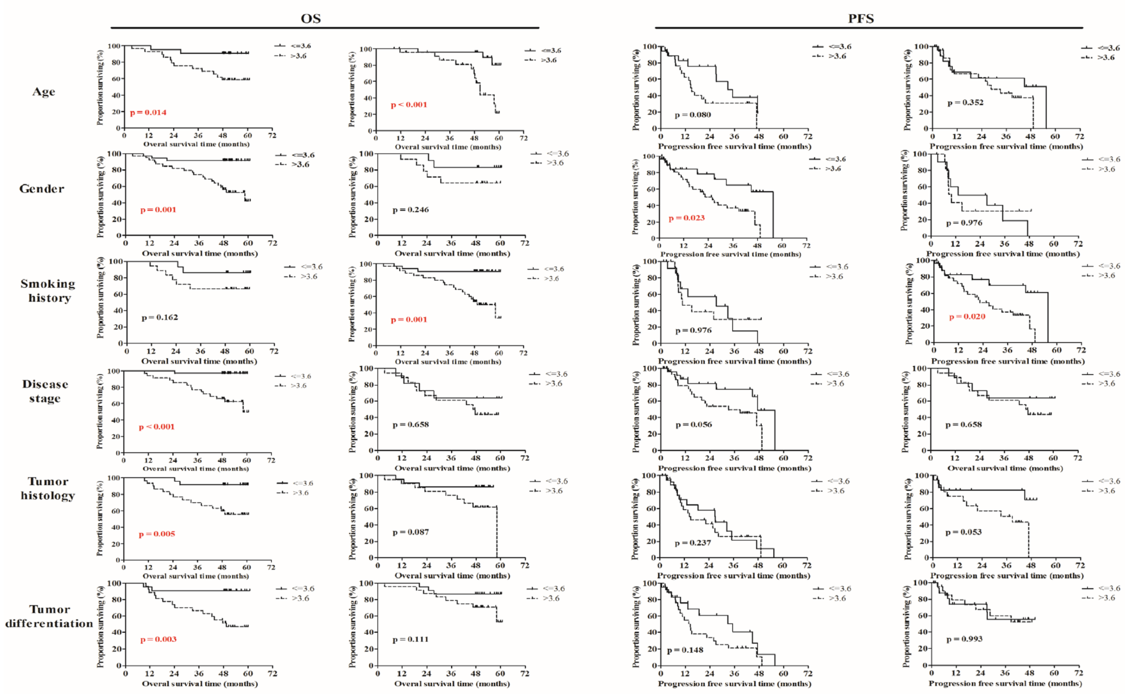

Supplementary Figure 1: Kaplan–Meier curves of OS and PFS for patients with low and high TMAR in sub-groups stratified according to clinicopathologic variables.

Supplementary Table 1: Distribution of patient characteristics between low and high TMAR groups

Clinicopathologic variables	Number (%)	TMAR[a]		
		Low (%) (n=47)	High (%) (n=53)	p value[b]
Age				
<60	51	22 (43)	29 (57)	0.321
>=60	49	25 (51)	24 (49)	
Gender				
Male	74	35 (47)	39 (53)	1.000
Female	26	12 (46)	14 (54)	
Smoking history				
Never	33	15 (45)	18 (55)	0.777
Prior or current	67	32 (48)	35 (52)	
Disease stage				
Early (stage I & II)	71	36 (51)	35 (49)	0.088
Advanced (stage III & IV)	29	11 (38)	18 (62)	
Tumor histology				
Adenocarcinoma	54	24 (44)	30 (56)	0.396
Squamous	43	22 (51)	21 (49)	
Others	3	1 (33)	2 (67)	
Tumor differentiation				
Poorly	49	22 (45)	27 (55)	
Moderately	47	23 (49)	24 (51)	0.575
Well	1	1		

[a] TMAR, tumor microvessel aspect ratio; Low TMAR <= 3.6, High TMAR > 3.6.
[b] p value was calculated using Fisher's exact test.
